# Chronic Implications of Bilateral Foot Pattern Variability in Schoolchildren

**DOI:** 10.3390/healthcare13202586

**Published:** 2025-10-14

**Authors:** Magdalena Rodica Traistaru, Mihai Cealicu, Daniela Matei, Miruna Andreiana Matei, Liliana Anghelina, Doru Stoica

**Affiliations:** 1 Faculty of Nursing, University of Medicine and Pharmacy Craiova, 200349 Craiova, Romania; rodica.traistaru@umfcv.ro (M.R.T.); liliana.anghelina@umfcv.ro (L.A.); 2 Faculty of Medicine, University of Medicine and Pharmacy Craiova, 200349 Craiova, Romania; mihaicealicu18@gmail.com; 3 Faculty of Medicine, University of Valencia, 46010 Valencia, Spain; andreian@alumni.uv.es; 4 Department of Theory and Methodology of Motor Activities, Faculty of Physical Education and Sport, University of Craiova, 200585 Craiova, Romania; doru.stoica@edu.ucv.ro

**Keywords:** foot morphology, gait symmetry, pediatric population

## Abstract

Background: Foot morphology plays a central role in musculoskeletal development during childhood. Variations in the medial longitudinal arch may influence walking mechanics, and excess body weight can further affect plantar structure and gait. Objective: This study examined the relationship between foot type, body mass index (BMI), and gait function in school-aged children, with particular focus on gait symmetry as a sensitive marker. Methods: Ninety-eight children aged 8–16 years were evaluated. Foot type was classified using a pressure platform, and gait was assessed with a wearable sensor. Outcomes included gait symmetry, walking speed, cadence, Timed Up and Go (TUG), and Six-Minute Walk Distance (6MWD). Results: Mixed bilateral foot patterns were observed in 46 of the 98 participants (47%). Significant associations were found between foot type, BMI, and gait symmetry (*p* < 0.01), while other mobility measures (speed, cadence, TUG, 6MWD) remained stable across groups. Children with normal bilateral feet showed the best gait symmetry, whereas mixed patterns had the lowest. Conclusions: Gait symmetry is a sensitive indicator of functional imbalance in schoolchildren and is strongly influenced by both foot morphology and body weight. Incorporating plantar assessment and BMI monitoring into routine pediatric evaluations may help clinicians identify children at risk for long-term musculoskeletal problems at an early stage.

## 1. Introduction

Human gait is a highly complex motor function that requires precise neuromuscular coordination. The foot is the main interface between the body and the ground, providing both shock absorption and stability [1,2]. The medial longitudinal arch plays a pivotal role, as its morphology influences plantar pressure distribution, lower limb alignment, and musculoskeletal efficiency [3,4]. Correct foot development during childhood is essential for postural control, locomotor efficiency, and overall musculoskeletal health [5]. Arch maturation continues into adolescence, and the timing of structural consolidation differs between boys and girls. [6,7,8,9,10,11].

Epidemiological studies highlight the high prevalence of foot arch variations in children. While approximately 60% exhibit normal plantar arches [12], up to 20–30% present either flatfoot or high-arched configurations [13,14]. Physiological flatfoot is common during the first decade of life, often asymptomatic and spontaneously corrected [15,16]. However, persistence of abnormal morphologies beyond this developmental window may generate progressive musculoskeletal alterations [17], leading to pain, reduced physical performance, and altered joint mechanics [18,19]. Pes cavus, though less frequent, is typically associated with muscular imbalance or neuromuscular conditions, becoming apparent before the age of 10 [20].

Such deviations disrupt the natural load distribution of the foot [21]. They also induce compensatory strategies at the ankle, knee, hip, and spine [22,23]. Recent meta-analyses confirm significant global variability in prevalence of pediatric flatfoot and link to risk factors such as age, sex, body mass index (BMI), activity level, and urban living [24], demonstrating broad epidemiologic relevance and justifying the focus on children.

Studies show that flexible flatfoot in school-aged children is associated with increased pain, reduced physical activity, and compromised biomechanics, thus underscoring the clinical importance of early identification [25]; additional research demonstrates altered physical performance in some tasks and highlights associations with balance/agility [26,27].

Foot morphology strongly influences gait symmetry, defined as the bilateral similarity of kinematic and kinetic parameters [28]. Symmetry is a recognized marker of neuromuscular control, efficiency, and postural stability. Recent advances in three-dimensional gait analysis and accelerometry have enabled detection of subtle asymmetries previously inaccessible to routine clinical examination [29,30]. Several indices have been developed, including the Symmetry Angle [31], Harmonic Ratio, and the Symmetry Index of the Center of Mass (SIBCoM), which provide quantitative assessment of gait balance [32,33]. However, their clinical applicability is limited by methodological variability, dependence on walking speed, and the lack of standardized reference data [34,35]. The literature reveals a scarcity of normative, standardized data for gait symmetry indices in children, especially in relation to foot morphology and this methodological gap limits their use in pediatric clinical practice.

In children, gait analysis presents specific challenges due to ongoing musculoskeletal maturation and dynamic changes in neuromotor control [36]. Since foot arch development coincides with gait refinement, pediatric populations offer a critical window for identifying structural–functional interactions and preventing long-term complications [37].

There is no established set of reference values for gait symmetry indices across different foot types in children; filling this void is essential for evidence-based screening and intervention planning.

Although extensive research has explored gait mechanics and symmetry in adults, there remains a critical gap in understanding how foot morphology affects locomotor function in pediatric populations. This knowledge is essential, as childhood represents a decisive period for both foot arch development and gait pattern consolidation.

Structural deviations of the medial longitudinal arch, even when asymptomatic, may predispose children to altered biomechanics, impaired physical performance, and long-term musculoskeletal complications if left unrecognized [36,37].

Moreover, despite the availability of advanced gait symmetry indices such as SIBCoM, standardized reference values for children with different foot morphologies are lacking, limiting their translation into daily clinical practice [32].

This study integrates gait symmetry analysis with functional performance testing. Together, these methods provide a framework to evaluate how foot structure influences locomotor efficiency in school-age children. Establishing these correlations will not only enhance early identification of at-risk populations but also contribute to the development of preventive and rehabilitative strategies tailored to pediatric needs.

By combining objective gait symmetry assessment with physical performance tests such as the Timed Up and Go (TUG) and the 6-Minute Walk Test (6-MWT), this study adopts a holistic methodological approach that may serve as a foundation for future interventional strategies in pediatric podiatry and rehabilitation.

The primary objective of the study was to classify foot morphology in active schoolchildren, distinguishing between flat, cavus, and normal arches, and to examine whether these structural variations are associated with differences in gait symmetry.

The secondary objectives included the evaluation of functional performance parameters (cadence, walking speed, TUG, and 6-MWT) in relation to foot morphology, as well as the identification of those indices most sensitive to structural variations in the pediatric foot.

## 2. Materials and Methods

### 2.1. Design Overview

This cross-sectional study was conducted between June 2024 and January 2025 at the Pediatrics and Physical Medicine and Rehabilitation Departments of Filantropia Clinical Hospital, Craiova. Each participant underwent a single evaluation session, consisting of anthropometric assessment, foot morphology classification, gait analysis, and physical performance testing.

**Figure 1:**
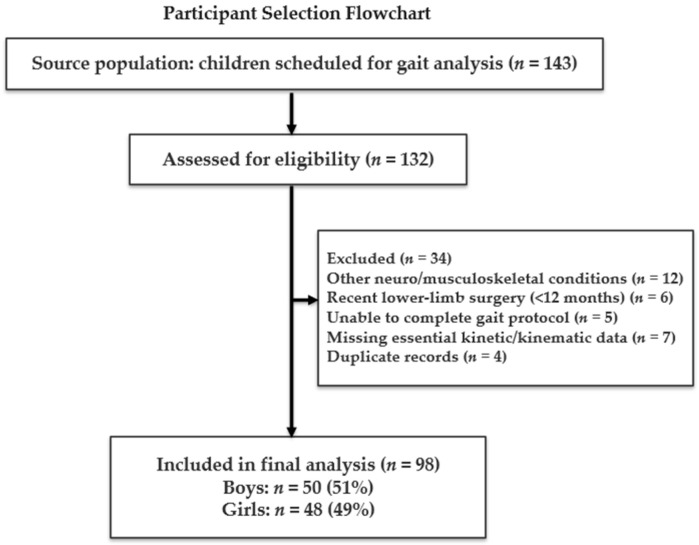
Diagram of study.

A total of 143 schoolchildren were scheduled for instrumented gait analysis, recruited through one pathway: consecutive sampling of children presenting at the hospital-based pediatric assessment program. Of the 143 eligible children, 132 provided informed consent to participate in the gait protocol (Figure 1). Thirty-four participants were excluded due to predefined reasons: neurological or musculoskeletal conditions (*n* = 12), recent lower-limb surgery (*n* = 6), inability to complete the protocol (*n* = 5), missing essential data (*n* = 7), or technical duplicate records (*n* = 4). The final cohort consisted of 98 children. The index date for each participant was defined as the date of their first completed gait analysis session.

All children were assessed using validated instruments, including anthropometric measurements, bilateral foot morphology, gait parameters (symmetry index, cadence, and walking speed), and physical performance tests (Six-Minute Walk Distance [6MWD] and TUG]).

Furthermore, the group was examined based on age and gender to confirm consistency and maintain the internal validity of the results.

Participants were eligible for inclusion if they met the following conditions: (a) provision of written informed consent from a parent or guardian and verbal assent from the child; (b) age between 8 and 16 years; (c) clinically healthy status confirmed through routine pediatric evaluation and review of medical records; (d) ability to understand and communicate in the national language; and (e) ability to walk independently and follow instructions.

### 2.2. Participants

Exclusion criteria were: (a) presence of psychiatric disorders confirmed by a specialist; (b) recent traumatic injuries; (c) diagnosed orthopedic, respiratory, cardiac, or neurological conditions, or congenital anomalies of the lower limb that could affect gait analysis; (d) foot pain or discomfort at the time of assessment; (e) history of lower limb surgery in the last six months; (f) current or recent (≤6 months) use of foot orthoses or orthopedic footwear; (g) neurodevelopmental disorders (e.g., autism spectrum disorder, severe ADHD) that could impede the correct execution of gait or performance tests; (h) chronic medication known to affect neuromuscular function, balance, or exercise capacity (e.g., antiepileptics, systemic corticosteroids); and (i) non-compliance or unwillingness to complete all functional tests. Extreme BMI categories (underweight or obesity) were not considered exclusion criteria; participants were retained in the study and stratified for analysis according to BMI classification.

The final study cohort comprised 98 children (50 boys and 48 girls). All participants were active schoolchildren, regularly involved in both curricular and extracurricular physical activities, most frequently football, basketball, or volleyball. Importantly, participation in sports was not an inclusion criterion but rather a descriptive characteristic of the group, ensuring comparability of baseline physical activity across participants. This selection was made to ensure homogeneity of exposure to standardized physical activity routines and to facilitate access to consistent testing conditions within the same institutional framework. Rural populations and children with sedentary lifestyles were not included, which reflects the characteristics of the urban pediatric population accessible to our center.

The age range of 8–16 years was deliberately selected to encompass critical developmental stages in childhood and adolescence. During this period, foot arch maturation occurs in parallel with gait refinement, neuromuscular coordination, and rapid somatic growth. Investigating foot morphology and functional parameters within this developmental window provides valuable insights into the interplay between structure and function, while also allowing for the early detection of gait abnormalities.

Eligible children and their parents or guardians were fully informed about the study objectives and procedures and provided with the necessary informed consent forms prior to participation.

### 2.3. Parameters and Measurements

Four categories of parameters were evaluated in all participants: anthropometric data, foot morphology, gait analysis parameters, and physical performance tests. All measurements were performed indoors, under standardized conditions (quiet environment, barefoot, light clothing), and at approximately the same time of day to reduce variability. Assessments followed the same sequence for all children: anthropometric evaluation, foot morphology, gait analysis, and functional performance tests. Sensor placement and calibration were consistently performed by the same examiner trained in pediatric gait assessment, to minimize inter-observer variability. Each test was performed twice, and if significant discrepancies were observed, a third trial was conducted; the best performance was retained for analysis.

Anthropometric Data: Height, weight, and body mass index (BMI = weight [kg]/height^2^ [m^2^]) were recorded. Weight was measured using an electronic scale (range 0–150 kg, accuracy 0.1 kg), and height was measured with a portable stadiometer (accuracy 0.1 cm). Measurements were performed with participants barefoot and wearing light clothing. Each measurement was repeated twice by the same examiner to ensure reproducibility.

**Figure 2:**
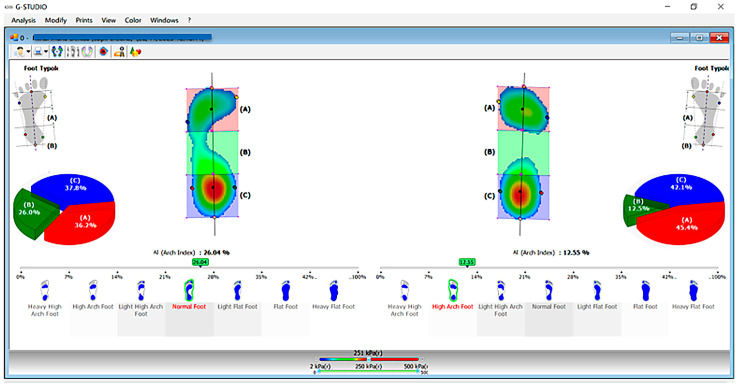
Example of the feet morphology (G-Studio software). The colored areas indicate anatomical regions and corresponding pressure distribution: red = forefoot (Zone A), green = midfoot (Zone B), and blue = heel (Zone C).

Foot morphology (Figure 2): Foot type and bilateral load distribution were assessed using the BTS P-WALK baro-resistive platform (BTS Bioengineering, Milan, Italy), integrated with the G-Studio software (BTS Bioengineering, Milan, Italy; available at: https://www.btsbioengineering.com). The device was calibrated immediately before testing in accordance with the manufacturer’s guidelines.

For this study, only the foot morphology classification was considered, establishing the bilateral foot pattern [38]. During assessment, participants stood barefoot in an upright position with arms alongside the body, feet approximately 5 cm apart, and gaze fixed forward. Each trial lasted 30 s, during which plantar pressures and arch configuration were recorded under evenly distributed load.

The definition of foot morphology followed the recorded measurements. We specify that the mixed foot pattern refers to a left–right asymmetry in foot morphology, assessed objectively using the BTS P-WALK baro-resistive platform (for example: right foot—normal, left foot—mild cavus). Gait analysis: Gait parameters were collected with the BTS G-WALK/G-SENSOR 2 wireless system (BTS Bioengineering, Milan, Italy). The sensor was secured with a dedicated belt over the sacrum, between the S1 and S2 vertebrae. Data were recorded while participants walked at their self-selected speed along a straight path of at least 7 m, ensuring a minimum of five complete gait cycles [39]. The system’s specifications and procedures are outlined in the BTS G-SENSOR 2 hardware manual (version 1.2.2, Document Number: ERGS2-01271-06, 2016) and the BTS G-WALK^®^ user manual (version 9.0.0, Document Number: ERGSN-01134-20, 2018).

The following parameters were analyzed: average walking speed (m/s), cadence (steps/min) and Symmetry Index (SIBCoM), which quantifies the ability to accelerate the center of mass similarly during right and left step cycles. A value closer to 100 indicates greater gait symmetry, with values above 90 generally found in non-pathological subjects [38].

The BTS G-WALK system has been validated for gait assessment in both adults and pediatric populations [40], ensuring reliability and reproducibility.

**Figure 3:**
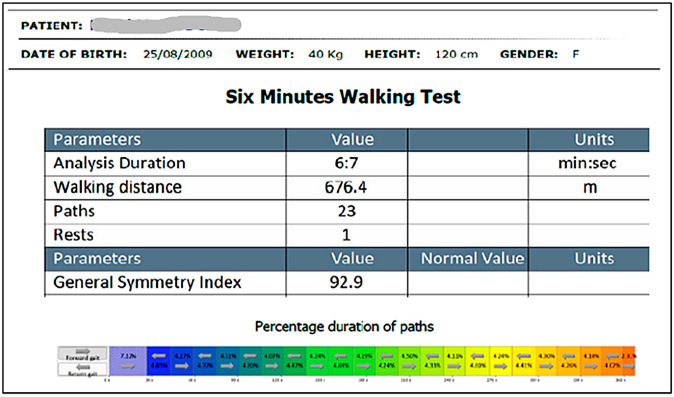
Example of 6MWT measurement (G-WALK software) The multicolored band visually represents the percentage duration of each gait phase; colors are for graphic differentiation only and have no physiological meaning.

**Figure 4:**
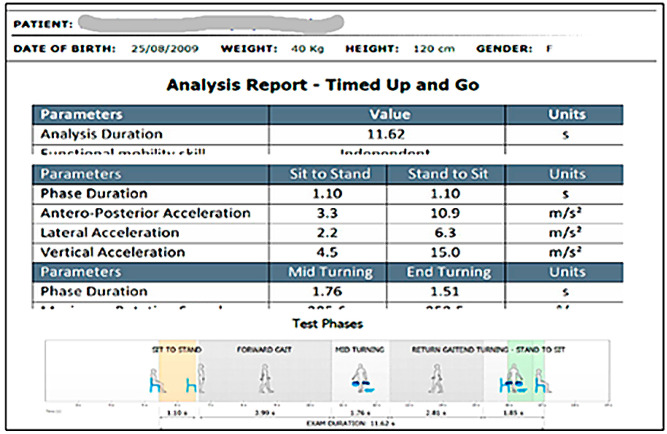
Example of TUG measurement (G-WALK software). The color bands represent the temporal distribution of each functional phase. Orange indicates the duration of the sit-to-stand transfer; light grey corresponds to the forward gait, mid-turning, and return phases; green represents the duration of the stand-to-sit transfer.

Physical Performance Tests: Functional capacity was assessed using two validated tests, both performed with the BTS G-WALK system, following standardized pediatric protocols:

- 6MWT (Figure 3): Conducted indoors along a 20 m corridor marked every 2 m. Children were instructed to walk as far as possible in six minutes. Standardized verbal encouragement and time reminders were given [41,42].
- TUG: Children started seated on a chair, then were instructed to stand, walk 3 m at a comfortable pace, turn, return, and sit down again. The total duration (seconds) was recorded [43] (Figure 4).

Both tests have been widely validated in pediatric populations [44,45] and provide robust measures of endurance, balance, and functional mobility.

Each child was given detailed instructions on how to perform the tests correctly and was familiarized with the equipment.

### 2.4. Ethics Approval

This study placed the highest priority on ensuring the safety and well-being of all participants, with particular attention to the fact that children represent a vulnerable population. Parents or legal guardians received detailed written information about the study’s objectives, potential benefits, risks, and confidentiality measures, in accordance with the General Data Protection Regulation (GDPR). Written informed consent was obtained from parents or guardians, while verbal age-appropriate assent was obtained from all children, after providing clear explanations adapted to their level of understanding.

Participants and their families were informed that participation was entirely voluntary and that they had the right to withdraw from the study at any point, without providing a reason or experiencing any negative consequences.

The study strictly followed the ethical principles outlined in the Declaration of Helsinki and Good Clinical Practice (GCP) guidelines. Ethical approval was obtained from the Ethics Committee of the University of Medicine and Pharmacy of Craiova (approval no. 38/1 March 2022). As this was a cross-sectional observational study without any intervention, trial registration in a public database was not required.

### 2.5. Statistical Analysis

Data were entered in Microsoft Excel and analyzed using SPSS version 26.0 (IBM Corp., Armonk, NY, USA) and Python libraries (pandas, NumPy, SciPy; available at: https://www.python.org).

- Descriptive statistics: for continuous variables, measures of central tendency (mean, median) and variability (standard deviation, minimum, maximum, interquartile range) were calculated. For categorical variables, absolute frequencies and percentages were reported. Data distribution was examined using skewness and kurtosis coefficients, and potential outliers were identified through quartile analysis.
- Comparative analysis: differences between groups defined by foot morphology (flat, cavus, normal arches) were assessed with the Kruskal–Wallis H test, appropriate for non-normally distributed data and unequal group sizes. Post hoc pairwise comparisons were performed using Dunn’s test with Bonferroni correction. Sex-based differences in foot morphology were evaluated using the Chi-square test, with sex coded numerically in the dataset (boys = 1, girls = 2).
- BMI-based analysis: participants were also stratified according to BMI category, with coding as follows: 1 = normal weight, 2 = overweight, 3 = obese. For each subgroup, descriptive statistics (mean, SD, min, max, IQR) were calculated, and cross-tabulations were performed to explore potential relationships between BMI and functional parameters.
- Correlation analysis: relationships between anthropometric, gait, and functional parameters were assessed using Spearman’s rank correlation coefficient, suitable for ordinal or non-normally distributed continuous data.
- Effect size and significance: effect sizes were calculated to complement significance testing (η^2^ for Kruskal–Wallis, Cramer’s V for categorical data, and r for correlations). A significance threshold of α = 0.05 was applied for all tests.

Justification of statistical choices. Parametric tests requiring normally distributed data (e.g., *t*-tests, ANOVA) were not applied, as several variables demonstrated non-normal distributions. Likewise, Shapiro–Wilk tests, 95% confidence intervals, and Levene’s test for homogeneity of variances were not used, since distributional assessment was based on skewness/kurtosis and the study design focused on non-parametric approaches. Linear regression analysis was also not applicable because the dependent variable (bilateral foot pattern) was categorical rather than continuous, and the applied non-parametric tests (Kruskal–Wallis and Dunn’s post hoc) were considered optimal for the study objectives.

## 3. Results

### 3.1. Baseline Characteristics

The baseline demographic and anthropometric characteristics of the study population are summarized in Table 1. Gender distribution was nearly equal, with 50 boys (51.0%) and 48 girls (49.0%), providing balanced representation for sex-based comparisons. All children resided in an urban environment, ensuring homogeneity of socio-geographic background within the cohort.

The mean age of participants was 10.8 ± 1.47 years, with a range of 8–16 years, reflecting a relatively homogeneous pediatric sample. with a comparable age distribution across sexes.

Mean BMI values were also similar between groups. Although girls showed slightly greater variability in both age and BMI, no substantial differences in central tendency were observed. The majority of children (*n* = 82, 83.7%) were classified as normal weight, while smaller groups were overweight (*n* = 9, 9.2%) and obese (*n* = 7, 7.1%).

### 3.2. Descriptive Statistics of Continuous Variables

Table 2 presents descriptive statistics for the main functional and gait parameters of the study cohort (*n* = 98). Functional performance measures showed stable distributions. TUG and 6MWD were approximately normally distributed. Gait parameters indicated high levels of locomotor performance. The mean Symmetry Index was 94.6% ± 2.9, with most participants clustered near the upper range, although a few outliers demonstrated more asymmetric patterns. Average walking speed was 1.15 ± 0.28 m/s, while average cadence was 117.0 ± 10.4 steps/min, both consistent with expected pediatric values.

### 3.3. Gender-Based Comparisons

Table 3 presents a comparative analysis of demographic, anthropometric, functional, and gait parameters between male (*n* = 50) and female (*n* = 48) participants. Functional performance measures showed slightly lower TUG times in boys (13.8 ± 2.2 s) compared with girls (14.5 ± 2.9 s), and a modestly greater six-minute walking distance (573.8 ± 73.8 m vs. 562.7 ± 67.6 m). Gait parameters indicated small sex-related differences. Girls demonstrated marginally higher gait symmetry (95.3 ± 2.7% vs. 94.0 ± 3.0%), whereas boys had slightly higher walking speed (1.16 ± 0.31 vs. 1.14 ± 0.24 m/s) and cadence (118.4 ± 10.1 vs. 115.6 ± 10.6 steps/min).

Overall, the results indicate minimal gender-based variation, with overlapping distributions and comparable variability across groups.

### 3.4. BMI-Based Comparisons

Table 4 summarizes the distribution of participants across BMI categories and their corresponding functional parameters.

Age increased progressively with BMI, from 10.6 ± 1.3 years in the normal-weight group to 12.7 ± 2.1 years in the obese group. As expected, BMI values differed distinctly between categories (normal: 18.4 ± 3.0 kg/m^2^; overweight: 26.3 ± 1.3 kg/m^2^; obese: 32.0 ± 1.4 kg/m^2^).

Functional performance showed comparable results across BMI categories. TUG times were stable (14.0–14.2 s), and six-minute walking distance (6MWD) ranged from 566 to 587 m, without significant differences between groups. Gait symmetry indices were consistently high, with mean values above 93% across all categories. Average walking speed and cadence showed no statistically significant variation by BMI group.

### 3.5. Foot Morphology Analysis

Table 5 presents the distribution of seven bilateral foot patterns in the study cohort (*n* = 98). The largest subgroup was the mixed pattern, present in 46 participants (47%). Normal bilateral arches were observed in 29 children (29.6%), while normal bilateral arches were observed in 7 children (7%). The other four types were described in a smaller number.

Functional parameters varied across foot patterns. Children with normal feet had the shortest TUG times (13.7 ± 2.1 s) and the highest symmetry index (96.1 ± 2.1%). The flat-foot group showed the lowest symmetry index (92.4 ± 3.7%), while the high-arch group achieved the greatest six-minute walk distance (6MWD: 588.3 ± 65.2 m). Across all patterns, mean walking speed ranged between 1.12 and 1.20 m/s, and cadence between 114 and 119 steps/min, with overlapping standard deviations.

**Figure 5:**
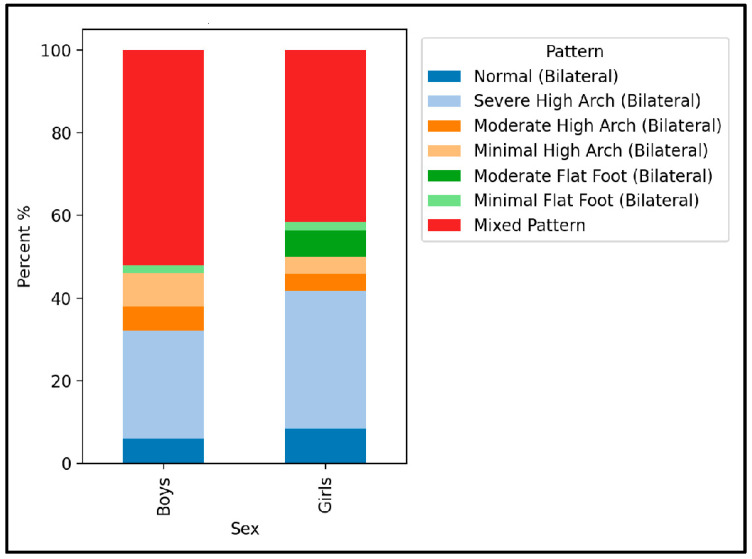
The distribution of feet pattern by gender.

Figure 5 illustrates the distribution of bilateral foot patterns stratified by sex. Mixed patterns were most frequent in both boys (44.0%) and girls (47.9%), followed by bilateral high-arch patterns (boys: 16.0%; girls: 12.5%). Normal bilateral arches were observed in 20.0% of boys and 16.7% of girls. The Chi-square test confirmed no significant differences in pattern distribution between sexes (χ^2^ = 6.15, *p* = 0.536), with a small effect size (Cramér’s V = 0.23).

**Figure 6:**
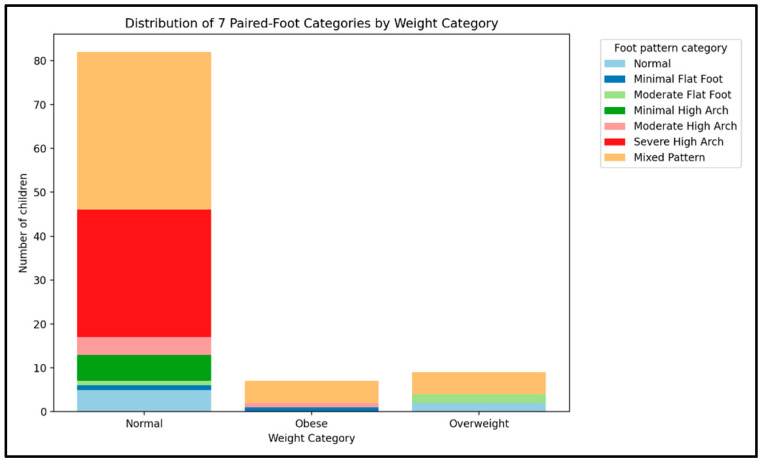
The distribution of feet pattern by BMI.

Figure 6 presents the distribution of foot pattern categories stratified by weight status. A statistically significant association was observed between weight category and foot pattern category (Chi-square test of independence, *p* = 0.0022).

Standardized residuals indicated the following deviations from expected frequencies:

- Overweight children: higher than expected prevalence of moderate flatfoot (residual ≈ +3.29).
- Obese children: higher than expected prevalence of minimal flatfoot (residual ≈ +2.27) and moderate high arch (residual ≈ +1.08).
- Normal-weight children: slightly higher frequencies of severe high arch (residual ≈ +0.96) and minimal high arch (residual ≈ +0.44).

Across all weight groups, the mixed pattern was consistently the most frequent classification, with residuals close to expected values (between −0.4 and +0.95). Flatfoot categories (minimal, moderate) appeared more often in higher-weight groups, while high-arch categories were relatively more represented among normal-weight children.

### 3.6. Correlation Analysis

Table 6 reports the results of the Kruskal–Wallis test performed to examine differences across bilateral foot pattern groups. Out of the seven studied parameters, two showed significant differences: BMI (H = 18.27, *p* = 0.0056) and Symmetry Index (H = 51.59, *p* < 0.0001). No significant differences were observed for age, TUG, 6MWD, average walking speed, or cadence (all *p* > 0.30).

**Figure 7:**
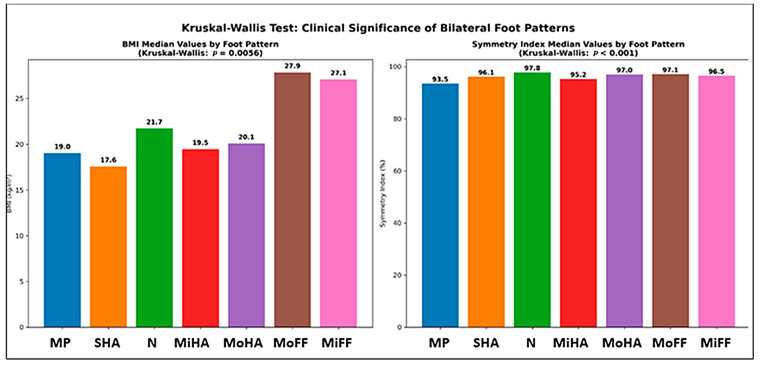
Kruskal–Wallis Test—Bilateral Foot Patterns (MP = mixed pattern, SHA = sever high arch, MoHA = moderate high arch, MiHA = minimal high arch, N = normal, MoFF = moderate flat foot, MiFF = minimal flat foot).

Post hoc Dunn analysis with Bonferroni correction confirmed that no pairwise comparisons were significant for BMI. For Symmetry Index, significant differences were observed between mixed patterns and bilateral moderate high-arch (*p* = 0.0049), as well as between mixed patterns and both bilateral normal (*p* < 0.0001) and bilateral severe high-arch feet (*p* < 0.0001) (Figure 7).

**Figure 8:**
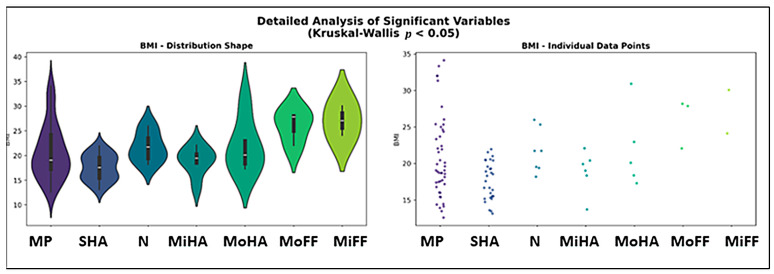
Detailed analysis of BMI—distribution depending on bilateral foot patterns (MP = mixed pattern, SHA = sever high arch, MoHA = moderate high arch, MiHA = minimal high arch, N = normal, MoFF = moderate flat foot, MiFF = minimal flat foot).

BMI distribution differed significantly between bilateral foot pattern groups (H = 18.27, *p* = 0.0056). Median BMI ranged from 17.6 kg/m^2^ in severe high-arch feet to 27.9 kg/m^2^ in moderate flat feet (Figure 8).

**Figure 9:**
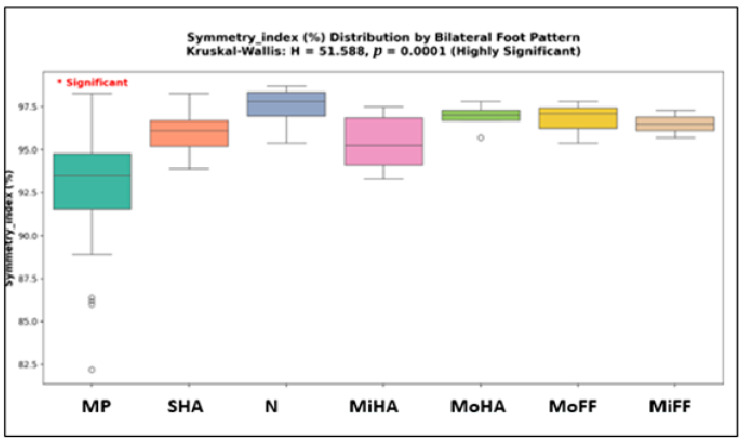
Detailed analysis of Symmetry Index—distribution depending on bilateral foot patterns (MP = mixed pattern, SHA = sever high arch, MoHA = moderate high arch, MiHA = minimal high arch, N = normal, MoFF = moderate flat foot, MiFF = minimal flat foot). * Significant = The Symmetry Index (SI) showed the strongest differences across foot patterns, differing significantly across the morphological patterns analyzed.

Symmetry Index showed the strongest between-group differences (H = 51.59, *p* < 0.001). Median values ranged from 93.5% in mixed patterns to 97.8% in normal bilateral arches (Figure 9).

No significant between-group differences were found for the other parameters analyzed: age (H = 2.23, *p* = 0.897), TUG (H = 5.92, *p* = 0.433), 6MWD (H = 7.17, *p* = 0.305), walking speed (H = 3.06, *p* = 0.802), and cadence (H = 4.40, *p* = 0.622). The distributions of these variables were broadly similar across foot pattern categories.

In summary, the Kruskal–Wallis and post hoc Dunn analyses confirmed that the Symmetry Index was the only parameter showing consistent and significant pairwise differences between foot pattern groups. Children with mixed patterns had significantly lower symmetry compared to all bilateral categories (*p* < 0.0001 vs. normal bilateral; *p* < 0.0001 vs. severe high-arch bilateral; *p* = 0.0049 vs. moderate high-arch bilateral). For BMI, overall group differences were significant (*p* = 0.0056), but no pairwise comparisons reached significance after Bonferroni correction. All other parameters, including age, TUG, 6MWD, average speed, and cadence, showed no significant differences across foot pattern groups.

### 3.7. Correlation Analysis (Spearman)

**Figure 10:**
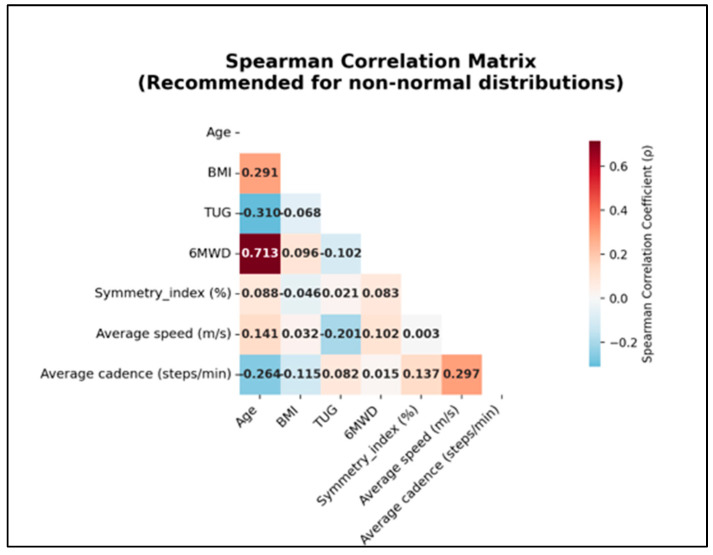
Spearman Correlation Matrix.

Spearman correlation analysis between the studied parameters is shown in Figure 10. A strong positive correlation was found between age and 6MWD (ρ = 0.713, *p* < 0.001). Age also correlated moderately and negatively with TUG (ρ = –0.310, *p* = 0.002), and weakly and positively with BMI (ρ = 0.291, *p* = 0.004). No significant correlations were observed between Symmetry Index and the other studied parameters.

## 4. Discussions

This study investigated the association between foot morphology, BMI, and functional gait parameters in school-aged children. Nearly half of the cohort displayed mixed bilateral patterns, indicating that plantar asymmetry is frequent in this developmental stage. Significant associations were identified between bilateral foot patterns, BMI, and gait symmetry, with the Symmetry Index emerging as the most sensitive functional indicator. In contrast, global measures such as TUG, 6MWD, walking speed, and cadence did not differ significantly across foot types, suggesting that these parameters are less discriminative in detecting subtle biomechanical variations.

The cohort showed a balanced gender distribution and a predominance of normal-weight children, supporting external validity for similar urban pediatric populations. Subgroup sizes for overweight and obese children were relatively small, which may limit statistical power for subgroup analyses [46,47,48]. Nevertheless, BMI stratification revealed graded differences across foot morphology categories: flatfoot patterns showed the highest BMI values, normal bilateral patterns clustered around healthy ranges, and mixed patterns demonstrated intermediate levels. A correlation between body weight and medial arch morphology was observed, although causality cannot be inferred. This association is consistent with reports of higher flatfoot prevalence in overweight children [49,50] and is reinforced by recent findings showing inflammation-related performance impairments in obese schoolchildren [51], as well as a meta-analysis confirming BMI as a strong predictor of abnormal plantar development [52]. The progressive increase in age across BMI categories further supports the role of maturational effects on body composition and gait biomechanics [53,54]. Our analysis confirmed that developmental asymmetries are relatively common in 8–16-year-olds, aligning with prior pediatric studies [55,56].

Flatfoot was relatively rare in our cohort, which contrasts with several reports in the literature [57]. A plausible explanation is the higher level of physical activity, as reduced activity is a strong risk factor for flatfoot [58]. Previous epidemiological studies in less active populations have consistently reported higher flatfoot prevalence [17,24,25,26]. Moreover, methodological differences such as the use of clinical indices (e.g., Clarke angle, FPI) versus dynamic plantar pressure platforms may partly account for the discrepancies observed [48]. Thus, both activity-related and methodological factors should be considered when interpreting the lower prevalence in our sample.

No significant differences in bilateral foot patterns were found between boys and girls, aligning with previous work showing minimal sex effects during pre- and early-puberty [59]. However, the literature remains inconsistent, and our null findings may also reflect limited statistical power to detect smaller sex-related effects, given subgroup sizes [60,61].

Methodological variability, including foot classification tools, thresholds, and unilateral versus bilateral approaches, likely contributes to these discrepancies [62]. Our use of computerized BTS P-Walk ensured reproducibility and transparency [41]. Previous reviews have highlighted the multifactorial nature of postural asymmetry and the need for standardized assessment [63].

In our study, most children demonstrated high gait symmetry, although some outliers showed reduced symmetry, consistent with reports that even healthy individuals display measurable asymmetries [34,64,65].

The Symmetry Index proved to be the most discriminative parameter: children with normal bilateral feet displayed the highest values, while those with mixed patterns demonstrated the lowest, forming a clear gradient. Post hoc Dunn analysis confirmed these findings, with significant pairwise differences between mixed and bilateral categories, underscoring the discriminatory power of the Symmetry Index compared with global parameters. Similar results have been reported in comparative gait studies, where symmetry indices consistently outperformed spatiotemporal measures [31,34,64].

Other global gait parameters, including walking speed and cadence, remained within normative values for age [66].

Sex differences in gait parameters were minimal and unlikely clinically relevant. Likewise, no significant group differences were observed for TUG or 6MWD, indicating that basic mobility and endurance are largely preserved across plantar categories. This aligns with prior research indicating that simple mobility tests may overlook subtler gait alterations in obese children [67].

From a clinical perspective, the frequent occurrence of mixed plantar patterns suggests potential value in early screening. However, in line with the cross-sectional design, these should be framed as implications for future interventional research rather than prescriptive recommendations.

Given the cross-sectional design, the following should be viewed as potential implications and directions for future interventional research rather than prescriptive recommendations: targeted plantar strengthening programs, individualized footwear guidance, and weight-management strategies. Future trials are needed to test whether these approaches can mitigate biomechanical imbalances and help preserve functional mobility.

These recommendations are supported by prospective rehabilitation studies showing improved performance and self-esteem in obese adolescents following kinesiotherapy [50] and by gait-focused interventions in overweight children [68].

The integration of digital gait analysis technologies into pediatric practice is also supported by pilot studies in athletes, where such systems captured subtle biomechanical deviations [69]. Our findings confirm their applicability to clinical pediatric populations. Furthermore, rehabilitation studies in older adults targeting sarcopenia, obesity, and chronic pain highlight the broader translational relevance of integrative approaches [70].

This study has several limitations. First, the relatively small cohort, particularly in overweight and obese groups, may have reduced statistical power for subgroup analyses. Second, the restriction of the sample to urban and physically active children may limit the generalizability of our findings. Results should therefore be interpreted with caution when extrapolated to rural or sedentary pediatric populations, who may present different patterns of foot morphology and gait characteristics. Third, the analysis relied exclusively on BTS P-Walk and G-Walk devices; although validated, complementary indices such as the Foot Posture Index, Clarke angle, or Wejsflog index were not included [71,72,73]. Fourth, other potentially relevant factors, including backpack use, footwear, and extracurricular activity, were not assessed, despite their known influence on plantar alignment [74]. The study did not employ multivariate regression models (e.g., logistic regression) to explore the combined effects of age, sex, and BMI. While non-parametric tests were appropriate for the sample size and distribution, the absence of regression analysis limits the ability to adjust for potential confounders. Finally, the cross-sectional design precludes causal inference.

Internal validity was supported by balanced sex representation, standardized protocols, repeated measurements, and objective digital tools that minimized observer bias. Non-parametric tests were appropriately applied for non-normal distributions, further supporting robustness.

External validity was enhanced by inclusion of a typical urban pediatric population with body composition largely consistent with national patterns. However, the limited number of overweight/obese participants and the exclusion of children with comorbidities restrict generalizability. The findings are therefore most applicable to otherwise healthy, physically active, urban schoolchildren. Larger and more diverse cohorts are needed to extend applicability.

## 5. Conclusions

In this cross-sectional study of school-aged children, bilateral foot patterns were found to be associated with BMI and gait symmetry, while other mobility parameters remained largely stable across groups. The Symmetry Index emerged as the most sensitive functional parameter for detecting differences between plantar patterns. These results highlight the importance of integrating plantar assessment and BMI monitoring in pediatric practice. However, due to the cross-sectional design, causal relationships cannot be established. The potential preventive value of early screening and intervention remains a hypothesis that should be confirmed in future longitudinal studies.

## Figures and Tables

**Table 1:** Baseline characteristics of study participants (*n* = 98).

Variable	Total (98)	Boys (50)	Girls (48)	*p*-Value
Age, years	10.8 ± 1.47 (8–16)	10.7 ± 1.30 (8–15)	10.9 ± 1.64 (8–16)	0.067
Sex, *n* (%)	98 (100%)	50 (51%)	48(49%)	0.078
BMI, kg/m^2^	20.1 ± 4.86 (12.58–34.13)	19.96 ± 4.53 (13.69–34.13)	20.29 ± 5.23 (12.58–32)	0.062
BMI categories, *n* (%)	98	50	48	
Normal weight	82 (83.7%)	45 (90%)	37 (77%)	
Overweight	9 (9.2%)	3 (6%)	6 (12.5%)	
Obese	7 (7.1%)	2 (4%)	5 (10.5%)	
Living environment	Urban: 98	Urban: 50	Urban: 48	

Data are presented as mean ± SD for continuous variables and *n* (%) for categorial variables. *p*-values based on the Kruskal–Wallis H test or Chi-square test (sex distribution).

**Table 2:** Descriptive Statistics for Continuous Variables.

Parameter	Count	Mean	Median	Std_Dev	Min	Max	Q1	Q3	Skewness	Kurtosis
TUG (seconds)	98	14.16	13.83	2.59	9.64	22.29	12.2	15.9	0.599	−0.026
6MWD (meters)	98	568.37	561.15	70.68	401.3	781.8	510.2	611.85	0.149	−0.157
Symmetry_ index (%)	98	94.61	95.05	2.91	82.2	98.7	93.55	96.68	−1.551	3.425
Average speed (m/s)	98	1.15	1.19	0.28	0.14	1.68	1.05	1.31	−1.367	3.298
Average cadence (steps/min)	98	117.03	115.75	10.4	90.7	145.7	110.6	124.15	0.216	−0.068

Data are presented as measures of central tendency (mean, median), variability (standard deviation, minimum, maximum, interquartile range) and potential outliers through quartile (Q1, Q3) analysis. Data distribution was examined using Skewness and Kurtosis coefficients.

**Table 3:** Gender-Based Comparative Analysis.

Parameter	Boys			Girls		
	N	Mean	SD	N	Mean	SD
TUG (seconds)	50	13.81	2.23	48	14.53	2.89
6MWD (meters)	50	573.79	73.81	48	562.73	67.58
Symmetry index (%)	50	93.99	3.01	48	95.26	2.69
Average speed (m/s)	50	1.16	0.31	48	1.14	0.24
Average cadence (steps/min)	50	118.44	10.13	48	115.57	10.57

Data are presented as measures of central tendency (mean) and variability (standard deviation).

**Table 4:** BMI Category-Based Analysis.

Parameter	BMI	N	Mean	SD	Min	Max
Age (years)	Normal	82	10.59	1.31	5	14
	Overweight	9	11.22	1.3	10	14
	Obese	7	12.71	2.06	10	16
BMI (kg/m^2^)	Normal	82	18.43	2.95	12.58	24.63
	Overweight	9	26.32	1.26	25	28.17
	Obese	7	31.97	1.39	30.07	34.13
TUG (seconds)	Normal	82	14.18	2.63	9.64	22.29
	Overweight	9	14.03	2.96	11.49	19.44
	Obese	7	14.15	1.9	11.15	16.51
6MWD (meters)	Normal	82	566.13	68.84	401.3	703.6
	Overweight	9	586.7	64.74	486.3	676.4
	Obese	7	571.07	103.09	500	781.8
Symmetry index (%)	Normal	82	94.59	2.72	82.2	98.7
	Overweight	9	95.91	2.35	91.3	98.6
	Obese	7	93.14	4.96	86	98.3
Average speed (m/s)	Normal	82	1.14	0.29	0.14	1.59
	Overweight	9	1.18	0.15	0.92	1.36
	Obese	7	1.26	0.32	0.86	1.68
Average cadence (steps/min)	Normal	82	117.39	9.66	97.4	140.1
	Overweight	9	119.12	9.7	107.1	139.5
	Obese	7	110.13	17.25	90.7	145.7

Data are presented as measures of central tendency (mean) and variability (standard deviation, minimum, maximum).

**Table 5:** Feet morphology correlations with anthropometric characteristics and functional parameters.

Feet_Pattern	Parameter	N	Mean	SD	Min	Max
Mixed Pattern	Age	46	10.89	1.54	9	16
	BMI	46	20.78	5.56	12.58	34.13
	TUG	46	14.25	2.27	9.64	19.44
	6MWD	46	565.39	72.5	415.6	781.8
	Symmetry index (%)	46	92.72	3.05	82.2	98.3
	Average speed (m/s)	46	1.15	0.25	0.22	1.68
	Average cadence (steps/min)	46	115.89	11.06	90.7	140.1
Severe High Arch (Bilateral)	Age	29	10.62	1.15	9	12
	BMI	29	17.55	2.61	13.13	21.95
	TUG	29	13.66	2.67	9.81	20.3
	6MWD	29	564.17	68.48	401.3	665.2
	Symmetry index (%)	29	96.01	1.22	93.9	98.3
	Average speed (m/s)	29	1.18	0.27	0.16	1.56
	Average cadence (steps/min)	29	118.5	9.08	102.1	137.5
Normal (Bilateral)	Age	7	10.86	0.38	10	11
	BMI	7	21.69	3	18.18	25.98
	TUG	7	14.18	2.03	11.49	17.78
	6MWD	7	592.96	61.97	501.6	660.4
	Symmetry index (%)	7	97.5	1.17	95.4	98.7
	Average speed (m/s)	7	1.19	0.2	0.95	1.41
	Average cadence (steps/min)	7	119.9	7.49	107.5	126.8
Minimal Flat Foot (Bilateral)	Age	2	10	2.83	8	12
	BMI	2	27.09	4.21	24.11	30.07
	TUG	2	13.3	1.7	12.1	14.5
	6MWD	2	480.45	27.65	460.9	500
	Symmetry index (%)	2	96.5	1.13	95.7	97.3
	Average speed (m/s)	2	1.3	0.33	1.06	1.53
	Average cadence (steps/min)	2	120.8	17.96	108.1	133.5
Minimal High Arch (Bilateral)	Age	6	11.5	1.52	10	14
	BMI	6	18.9	2.86	13.69	22.08
	TUG	6	13.11	2.34	10.3	16.65
	6MWD	6	581.78	97.7	471.6	703.6
	Symmetry index (%)	6	95.4	1.74	93.3	97.5
	Average speed (m/s)	6	1.2	0.26	0.86	1.57
	Average cadence (steps/min)	6	110.65	9.22	101.3	121.2
Moderate High Arch (Bilateral)	Age	5	11	1	10	12
	BMI	5	21.92	5.47	17.29	30.92
	TUG	5	16.3	4.17	10.65	22.29
	6MWD	5	605	45.58	533.3	654.2
	Symmetry index (%)	5	96.9	0.78	95.7	97.8
	Average speed (m/s)	5	1.02	0.45	0.43	1.67
	Average cadence (steps/min)	5	121.14	15.2	107.5	145.7
Moderate Flat Foot (Bilateral)	Age	3	9.67	4.04	5	12
	BMI	3	26.03	3.44	22.06	28.17
	TUG	3	16.82	4.1	12.19	19.97
	6MWD	3	568.07	65.16	493.1	611.1
	Symmetry index (%)	3	96.77	1.23	95.4	97.8
	Average speed (m/s)	3	0.88	0.64	0.14	1.28
	Average cadence (steps/min)	3	117.13	7.96	110.6	126

**Table 6:** Statistical analysis (Kruskal–Wallis test) between feet pattern and studied parameters.

	H-Statistic	*p*-Value	Significant
Age (years)	2.2295	0.8974	no
BMI (kg/m^2^)	18.2732	0.0056	yes
TUG (seconds)	5.9175	0.4325	no
6MWD (meters)	7.1702	0.3054	no
Symmetry index (%)	51.5881	0.0000	yes
Average speed (m/s)	3.0578	0.8016	no
Average cadence (steps/min)	4.4042	0.6222	no

## Data Availability

The data presented in this study are available on request from the corresponding author due to privacy and confidentiality concerns related to human subjects andethical restrictions imposed by the ethics committee
